# Dual-gated volumetric modulated arc therapy

**DOI:** 10.1186/1748-717X-9-209

**Published:** 2014-09-25

**Authors:** Benjamin Fahimian, Junqing Wu, Huanmei Wu, Sarah Geneser, Lei Xing

**Affiliations:** Department of Radiation Oncology, Stanford University, Stanford, CA USA; School of Health Sciences, Purdue University, West Lafayette, IN USA; Purdue School of Engineering and Technology, Indiana University School of Informatics, IUPUI, Indianapolis, IN USA

**Keywords:** Dual gating, IGRT, VMAT, SBRT

## Abstract

**Background:**

Gated Volumetric Modulated Arc Therapy (VMAT) is an emerging radiation therapy modality for treatment of tumors affected by respiratory motion. However, gating significantly prolongs the treatment time, as delivery is only activated during a single respiratory phase. To enhance the efficiency of gated VMAT delivery, a novel dual-gated VMAT (DG-VMAT) technique, in which delivery is executed at both exhale and inhale phases in a given arc rotation, is developed and experimentally evaluated.

**Methods:**

Arc delivery at two phases is realized by sequentially interleaving control points consisting of MUs, MLC sequences, and angles of VMAT plans generated at the exhale and inhale phases. Dual-gated delivery is initiated when a respiration gating signal enters the exhale window; when the exhale delivery concludes, the beam turns off and the gantry rolls back to the starting position for the inhale window. The process is then repeated until both inhale and exhale arcs are fully delivered. DG-VMAT plan delivery accuracy was assessed using a pinpoint chamber and diode array phantom undergoing programmed motion.

**Results:**

DG-VMAT delivery was experimentally implemented through custom XML scripting in Varian’s TrueBeam™ STx Developer Mode. Relative to single gated delivery at exhale, the treatment time was improved by 95.5% for a sinusoidal breathing pattern. The pinpoint chamber dose measurement agreed with the calculated dose within 0.7%. For the DG-VMAT delivery, 97.5% of the diode array measurements passed the 3%/3 mm gamma criterion.

**Conclusions:**

The feasibility of DG-VMAT delivery scheme has been experimentally demonstrated for the first time. By leveraging the stability and natural pauses that occur at end-inspiration and end-exhalation, DG-VMAT provides a practical method for enhancing gated delivery efficiency by up to a factor of two.

## Background

Respiratory induced tumor motion is the major complicating factor in radiotherapy of thoracic and upper abdominal targets. A variety of techniques have been developed for the clinical management of organ motion, each with distinct advantages and drawbacks [1–5]. These techniques can be generally categorized in order of approximate increased technical complexity as motion encompassing irradiation, breath-hold methods, compression methods, gating methods, and dynamic tracking methods. Among these, gating methods have gained clinical traction as they limit the volume of normal tissue irradiated relative to motion encompassing irradiation, yet provide a reliable and a technical feasible alternative to continuous tracking irradiation [6–11].

With improvements in dynamic delivery, Volumetric Modulated Arc Therapy (VMAT) has emerged as an efficient alternative to static field Intensity Modulated Radiotherapy (IMRT) for producing highly conformal dose distributions [12–14]. More recently, gated VMAT has been implemented for treating tumors influenced by respiratory motion by restricting the arc delivery to a single stable portion of the respiratory cycle, such as the end-of-exhale (EOE) phase. While gated VMAT is a promising technique, since the delivery is restricted to a narrow segment of the respiration, the treatment time is significantly increased, which depending on the width of the gating window, can result as much as 5.5 times relative elongation of the treatment time relative to non-gated treatments [15, 16]. Prolonged treatment time can result in dosimetric inaccuracy, baseline shift, increased patient discomfort, degradation of radiobiological efficacy, and reduction of clinical throughput. Moreover, improving gated delivery efficiency is of particular importance in Stereotactic Body Radiation Therapy (SBRT), because of the already protracted delivery associated with large dose fractions. In general, gating window selection is a compromise between the residual tumor movement within the gating window and dose delivery time. Because there is generally relatively little residual tumor motion within the end-of-exhale (EOE) phase [7], the EOE window is often selected for gating. However, the end-of-inhale (EOI) phase is also relatively stable, and can be dosimetrically advantageous in certain cases [8–10]. A delivery scheme that enables beam-on during both EOE and EOI would make it possible to combine the delivery advantages of both phases. It is noted that, while theoretically, continuous tracking delivery has the potential for full duty cycle efficiency [17], due to stringent technical demands, and more importantly, clinical concerns in accurately predicting and irradiating the more variable portions of the breathing cycle in-between the more stable exhale and inhale, its clinical application on a conventional multi-leaf collimator linac has been limited. For these reasons, a simple and technically practical solution for enhancing gated delivery that irradiates only the clinically reliable portions of the breathing cycle is desirable.

To address the limitations in efficiency of gated VMAT, in this work we introduce and demonstrate Dual-Gated VMAT (DG-VMAT) – a method that alternatively delivers dose at both the inhale and exhale phases during a VMAT delivery. While gating on a single phase is currently implementable on most modern linacs gating on more than a single phase requires the synchronization of the MLCs and gantry motion to the different locations of the target at the two different phases of the respiratory cycle. A practical approach to such an implementation is presented, and the feasibility is demonstrated by experimentally implementing DG-VMAT on linac using custom scripting.

## Methods

### DG-VMAT delivery scheme

**Figure 1:**
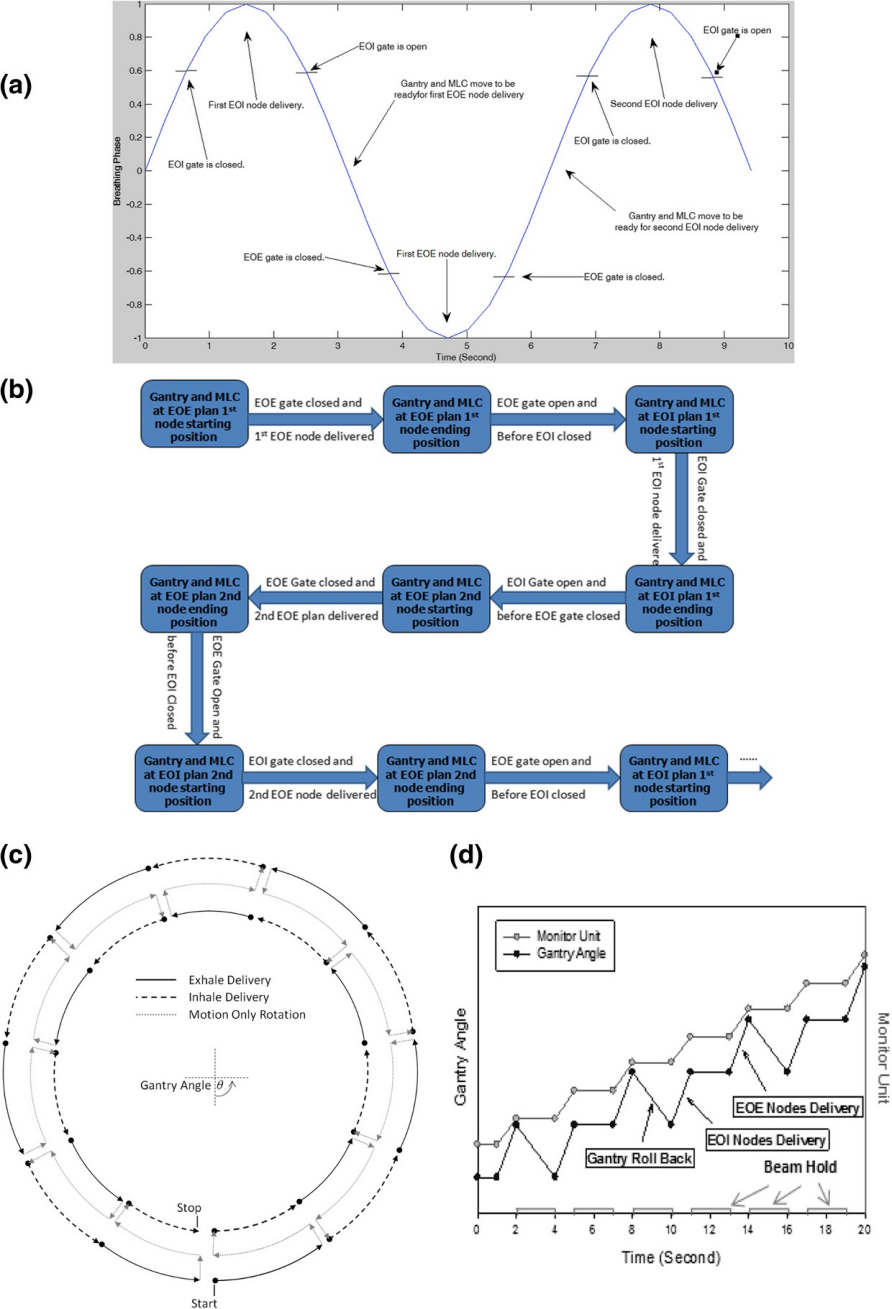
**Cell proliferation measurements of MDA-MB-231 cells for control and LMMS groups assessed with a)** Trypan Blue dye exclusion method **b)** MTT assay. **c)** Number of MDA-MB-231 cells that were detached from the plastic counted by Trypan Blue stain. **d)** Cell proliferation of MCF10A cells assessed with MTT assay. (*: p < 0.05 between LMMS and sham controls).

The design of DG-VMAT is complicated by the fact that both the MLC motion and the gantry motion must be coupled and synchronized with two different portions of the respiratory cycle. A practical solution to this is provided through the scheme shown in Figure 1. Given two optimized plans corresponding separately to the exhale (EOE) and inhale phases (EOI) of a representative trace in Figure 1a, the dual-gated rotational arc delivery, as described in the flowchart of Figure 1b, starts from the initial node of one of the plans, taken here as the EOE plan. After the initial EOE arc delivery segment, the beam is turned off and the linac gantry rotates back with the MLC assuming the starting position specified in the EOI plan in preparation for the delivery. The first EOI node is delivered, and since the gantry position at completion of the first EOI gate is the same as the starting position of the EOE window of the second breathing cycle, the gantry remains stationary during the transition from EOI to EOE delivery. The alternating delivery process continues until all the nodes or MUs are delivered. The resulting gantry motion is demonstrated in Figure 1c, which highlights the rolling back and forth of the gantry for the alternating delivery of the EOE and EOI phases. The behavior of the gantry motion and its relation to the beam delivery, as defined by the cumulative monitor units, is plotted as function of time in Figure 1d.

### Treatment planning

The method necessitates two independent plans to be optimized at the EOE and EOI phases using a 4DCT simulation scan. In the feasibility study, an SBRT patient case with a 4D planning CT and approximately 2 cm superior-inferior tumor motion between EOE and EOI was selected to demonstrate the DG-VMAT planning and delivery process. Two independent VMAT treatment plans were generated using Varian’s Eclipse treatment planning system (Version 8.9); one for EOE and the other for EOI, based on the respective exhale and inhale phase images of the 4DCT scans. For each of the two plans, a dose of 15 Gy was prescribed to the 95% of PTV volume, and a full arc plan consisting of 177 segments (control points) was optimized for the same dose prescription and arc optimization criteria. 10 MV Flattering Filter Free (FFF) beams were used for treatment planning. A fractionation scheme 15 Gy in 3 was set for delivery.

### Experimental implementation and dosimetric validation

DG-VMAT plan scheme in Figure 1 was experimentally implemented for an SBRT plan using Developer Mode XML scripting in the TrueBeam™ STx platform (Varian Medical Systems, Palo Alto, CA), which enables programmed control of all system parameters. Using control points specifying the MLC leaf sequences and MUs derived from the independent single phase plans, a DG-VMAT delivery was programmed by sequentially interleaving the control points of the EOE and EOI plans, and enabling beam-on at the two corresponding phases of the respiratory surrogate phase.

Dual-gated VMAT plans were delivered and validated by running the formulated XML script, triggered by the RPM infrared camera and reflector system affixed to the motion platform (Varian Medical Systems, Palo Alto, CA). The delivered dose was measured by using a 0.015 cc PTW N31014 pinpoint chamber (PTW, Freiburg, Germany) in 14 cm of solid water and a Delta 4 diode array (ScandiDos, Uppsala, Sweden). Both the pinpoint chamber and Delta 4 phantom were placed on top of a motion platform, which served to simulate breathing motion. A 2 cm motion in the superior-inferior direction with a period of 6 seconds was utilized, with the inhale and exhale gating windows set at 25% of the full period. The measured dual-gated dose distribution was compared to the summed inhale and exhale dose distributions computed using an Eclipse AAA version 8.9.08. The delivery time was measured and compared to conventional single gated delivery, corresponding to the EOE plan scaled to an equivalent total dose.

## Results

**Figure 2:**
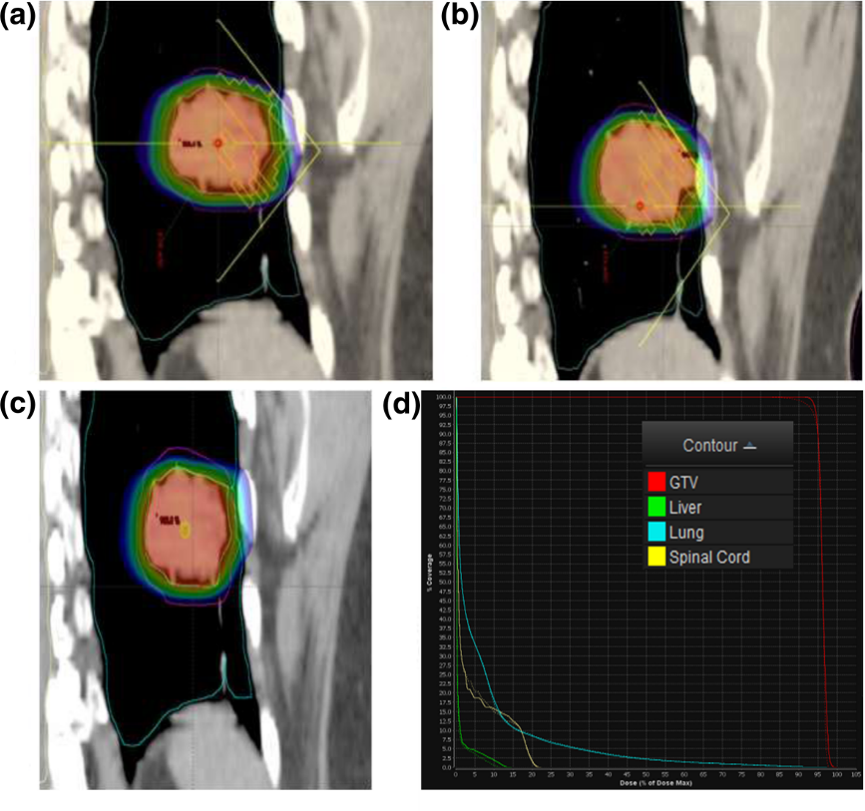
**Isodose distributions of individual (a) EOE, (b) EOI, and (c) summed dual-gated plan. (d)** Dose-volume histograms of the single EOE gating plan (solid line) and deformed dose summation of the dual-gated plan (dashed line).

Figure 2a and 2b show the dose distributions for the EOE and EOI plans, respectively. A composite plan, consisting of the EOI plan deformed doses imposed onto EOE CT images and summed with EOE plan dose, is presented in Figure 2c. The dose-volume histograms (DVHs) for the conventional exhale-gated plans (solid lines), and the dual-gated plan (dashed lines), are depicted in Figure 2d as computed using MIM imaging fusion software (MIM, Cleveland, OH). It is noted that there is some variation in the coverage of the EOE and EOI plans, which in addition to the errors and artifact of the dose warping and image registration process, results in variation in the estimated final DVHs [18]. In general, the dosimetric variation of DG-VMAT versus conventional single gated plans will depend on the patient anatomy on EOE and EOI phases, and the quality of the plans for each phase.

**Figure 3:**
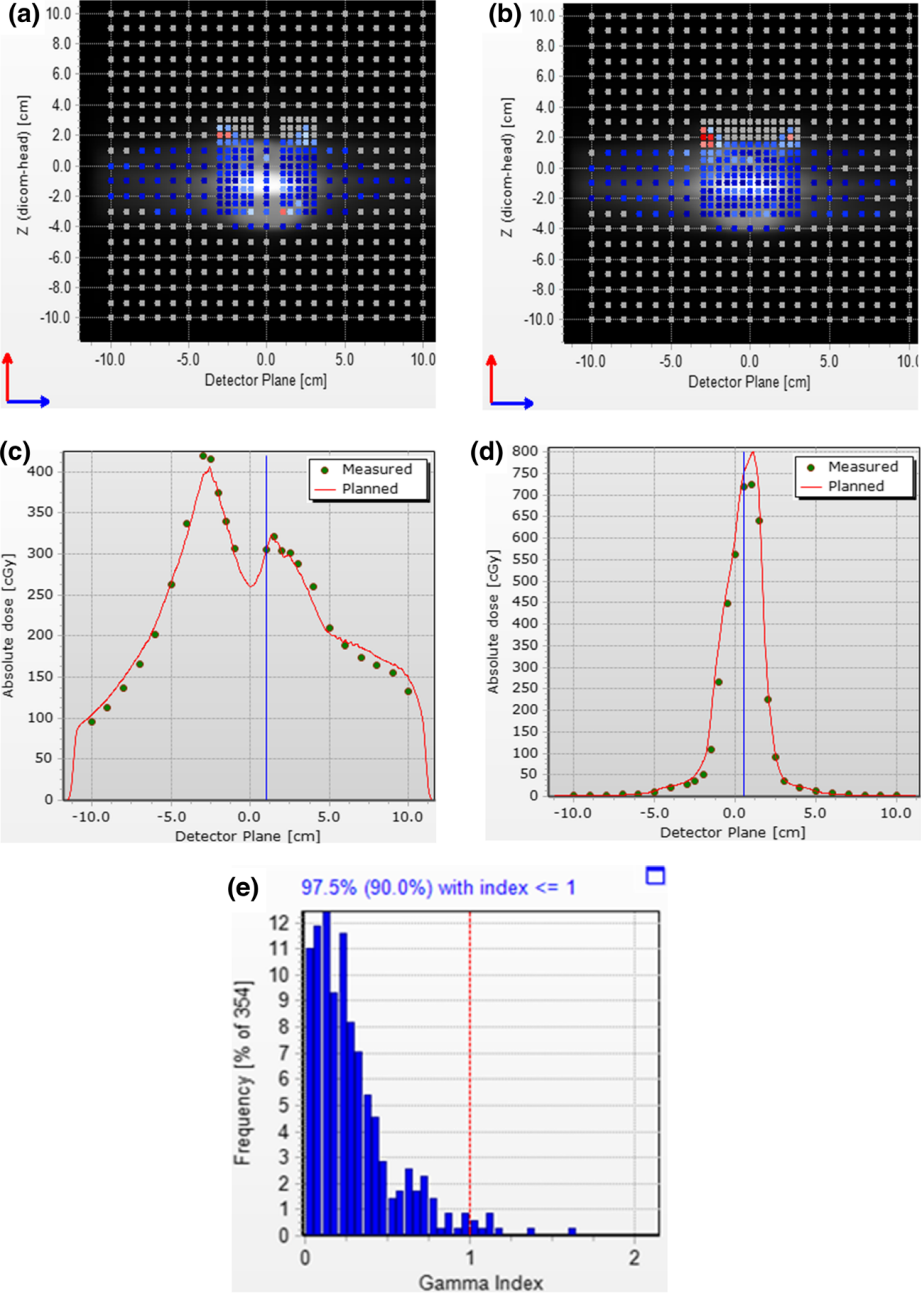
**Dosimetric validation of DG-VMAT using a diode array (a), (b) Display of the diodes passing the 3%/3 mm gamma test for the two crossed plane diode arrays (c) Horizontal dose profile of dose plane in (a), (d) Vertical dose profile of dose plane in (b), (e) Histogram of the gamma distribution.**

Figure 3 displays the dosimetric comparison of delivery, relative to the planned DG-VMAT plan using the diode array. The deviations as analyzed using the a 3 mm/3% gamma-test criterion; Figure 3a,b displays the passing points in the two planes of the diode array, while the dose profiles and the gamma histogram is shown in Figure 3c-e. For the dual-gated delivery, 97.5% of the measurement points pass the gamma-test criterion a gamma < 1. As indicated by Figure 3a,b, the failed points were at the primarily at periphery of the field in low dose regions. In addition to the diode measurements, the pinpoint chamber absolute dose measurement agreed with the dose calculation within 0.7%.

The delivery time reduction was assessed through comparison of the DG-VMAT delivery time with that of the conventional EOE plan scaled to the same dose. The conventional EOE gated VMAT delivery requires 346 seconds for the studied case, while the proposed DG-VMAT technique is delivered in 177 seconds per fraction. Thus, for this particular case, dual-gated VMAT provides a 95.5% improvement in delivery efficiency compared to the corresponding single-gated delivery.

## Discussion

Implementation of DG-VMAT requires the synchronization of the gantry motion and MLC with two phases of the respiratory cycle. As such, for half the transitions between exhale and inhale phases, the gantry is required to roll back between the phases, as depicted in Figure 1c,d. This is shown to be possible with the TrueBeam™ STx, which has a gantry rotation speed of 6 degrees/second, and a MLC leaf speed of 2.5 cm/second at isocenter. The DG-VMAT delivery required a roll-back of an average of 2.05 degrees, which is achieved in 0.34 seconds. Since the transition time between exhale and inhale gating windows was 1.5 seconds, there was more than sufficient time for the gantry and MLC to move to the planned positions in preparation for the subsequent nodal delivery. Considering an average breathing cycle of 4–6 seconds, such a motion is within the limits of current linacs as demonstrated in this first experimental demonstration.

**Figure 4:**
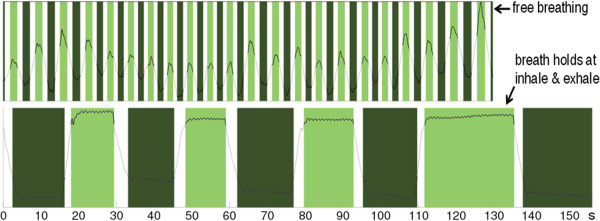
**Dual-gated dynamics of a healthy individual under free breathing (top) and coached inhale and exhale breath-holds (bottom).** The inhale and exhale gating windows are indicated by the light and dark green windows, respectively.

While the results indicate that the treatment time may be reduced by nearly a factor two for an ideal breathing pattern, a number of issues must be considered for implementation in a clinical setting. Most importantly is the variability in breathing patterns of human subjects. Specifically, it is known that under free breathing, subjects may spend more time in the exhale phase than inhale. If un-coached, a reduction of the magnitude of the efficiency enhancement with dual gating may be expected. In the current implementation of DG-VMAT, it is explicitly assumed that there is 1:1 ratio between the EOI and EOE gating window. To achieve this during a patient treatment, coaching via audio-visual guidance must be used. Specifically, the patient will be directed to briefly hold their breath at inhale and exhale for equal time intervals that are known from a simulation study to be comfortably tolerable for the patient. Through audio-visual guidance, the patient will be instructed on when to exit the EOI or EOE phase to proceed to the next delivery node. Such a technique has been experimentally shown to effectively equalize the inhale and exhale phases in healthy human subjects by Geneser *et al.*[19], as shown in Figure 4.

In this initial work, treatment planning was performed with the inhale and exhale phase optimized independently of each other. 4D treatment planning [20–22] may be adapted for a more cohesive optimization of the two phases through which DVH parameters are simultaneously optimized.

Several observations on the limitations and advantages of DG-VMAT can be made in relation to other respiratory management techniques. DG-VMAT is technically more complex than breath-hold techniques. Deep inspiration breath hold may achieve more advantageous anatomical separation for normal tissue sparing and has become more feasible with the use of high dose-rate flattening-filter-free beams [23, 24]. However, prolonged breath holding, as required to deliver SBRT doses, may not be tolerated by portions of the patient population, specifically those with already compromised lung function. Gating presents an alternative solution for such patients. Gating however, inherently results in significantly higher total treatment times due the fact the beam is conventionally activated for one phase of the breathing cycle. Dual gating, aims to enhance the efficiency of gating. While the technical complexity for such a delivery is higher than conventional gating, it represents significant simplification of alternate dynamic tracking proposals. More importantly, relative to tracking, dual gating only utilizes the stable portions of the respiratory cycle, and thereby eliminates intermediate irradiation between exhale and inhale which is known to be unstable and unpredictable.

## Conclusions

To enhance the delivery efficiency of gated VMAT, a technique for dual-gated delivery, leveraging the natural pauses that occur at peak-inspiration and exhalation for irradiation, has been proposed. The technique which necessarily coordinates the gantry rotation and MLC modulation with two different phases of respiratory cycle was experimentally implemented using custom XML programing in TrueBeam™ STx Developer Mode. The results presented herein demonstrate the first successful delivery of DG-VMAT which is shown to result in nearly a doubling of treatment delivery efficiency for ideal sinusoidal respiratory motion. For clinical implementation on patients, audio-visual guidance may be used to coordinate the breathing with the delivery. Dual-gated delivery efficiency can be further improved with additional linac hardware and software modifications to enable implementation in clinical mode. As compared to the existing respiratory-gating VMAT technique, a major advantage of DG-VMAT is that it substantially reduces treatment duration with a modest but practically achievable increase in complexity of the treatment delivery processes. DG-VMAT can potentially provide a compromise between breath-hold, gating, and tracking techniques by increasing the tolerability relative to breath-hold, reducing technical demand and potential inaccuracies associated with irradiation of variable portions of the respiratory cycle relative to tracking techniques, and increasing the efficiency of treatment relative to conventional single window gating.

## Authors’ information

Benjamin Fahimian and Junqing Wu are co-first author.
